# Hydrogen Sulfide Responsive Phototherapy Agents: Design Strategies and Biological Applications

**DOI:** 10.1021/acsbiomedchemau.3c00028

**Published:** 2023-06-23

**Authors:** Musa Dirak, Sarp E. Turan, Safacan Kolemen

**Affiliations:** † Koç University, Department of Chemistry, 34450 Istanbul, Turkey; ‡ Koç University Research Center for Translational Medicine (KUTTAM), 34450 Istanbul, Turkey

**Keywords:** cancer, phototherapy, bioimaging, theranostics, hydrogen sulfide, photosensitizers, activity-based agents, multimodal therapy

## Abstract

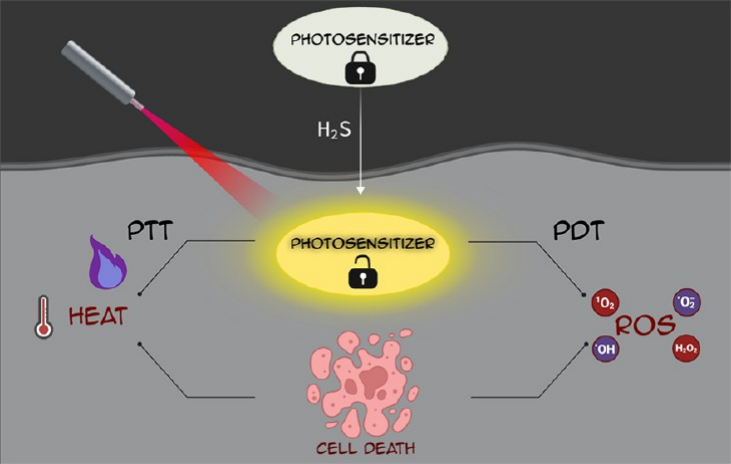

Hydrogen sulfide (H_2_S) is one of the critical gasotransmitters, which play important roles in regular physiological processes, especially in vital signaling pathways. However, fluctuations in endogenous H_2_S concentration can be linked to serious health problems, such as neurodegenerative diseases, cancer, diabetes, inflammation, cardiovascular diseases, and hypertension. Thus, it has attracted a great deal of attention in therapeutic applications, specifically in the field of phototherapy. Photodynamic therapy (PDT) and photothermal therapy (PTT) are two subclasses of phototherapy, which utilize either reactive oxygen species (ROS) or local temperature increase upon irradiation of a photosensitizer (PS) to realize the therapeutic action. Phototherapies offer unique advantages compared to conventional methods; thus, they are highly promising and popular. One of the design principles followed in new generation PSs is to build activity-based PSs, which stay inactive before getting activated by disease-associated stimuli. These activatable PSs dramatically improve the selectivity and efficacy of the therapy. In this review, we summarize small molecule and nanomaterial-based PDT and PTT agents that are activated selectively by H_2_S to initiate their cytotoxic effect. We incorporate single mode PDT and PTT agents along with synergistic and/or multimodal photosensitizers that can combine more than one therapeutic approach. Additionally, H_2_S-responsive theranostic agents, which offer therapy and imaging at the same time, are highlighted. Design approaches, working principles, and biological applications for each example are discussed in detail.

## 1 Introduction

### 1.1 Hydrogen Sulfide (H_2_S)

Hydrogen sulfide (H_2_S), a colorless gas with an unpleasant odor, has been widely identified as a toxic substance for decades.^1−6^ After H_2_S was recognized as the third gasotransmitter, in addition to carbon monoxide (CO) and nitric oxide (NO), its importance in biological systems was unveiled.^7−13^ H_2_S can be produced through enzymatic or nonenzymatic pathways.^14,15^ Enzymatic pathways begin with the conversion of homocysteine to cystathionine by the catalytic action of cystathionine β-synthase (CBS). Subsequent conversion of cystathionine to cysteine then takes place by cystathionine γ-lyase (CSE). Cysteine can finally be converted to H_2_S together with NH_4_^+^ and pyruvate. While this pathway takes place in the cytosol, cysteine can be converted to 3-mercaptopyruvate by aspartate aminotransferase (AAT) in mitochondria, which is subsequently transformed to H_2_S by 3-mercaptopyruvate sulfurtransferase (MPST).^2,4,15,16^ The nonenzymatic production pathways of endogenous H_2_S are not well-defined. However, recent studies have demonstrated that one route utilizes the catalytic action of iron and vitamin B6, while cysteine acts as a substrate.^17^ H_2_S can also be generated from polysulfides present in garlic by human red blood^18^ cells or from thiosulfate under hypoxic conditions,^19^ but these pathways leading to H_2_S generation still require further investigation.

**Figure 1:**
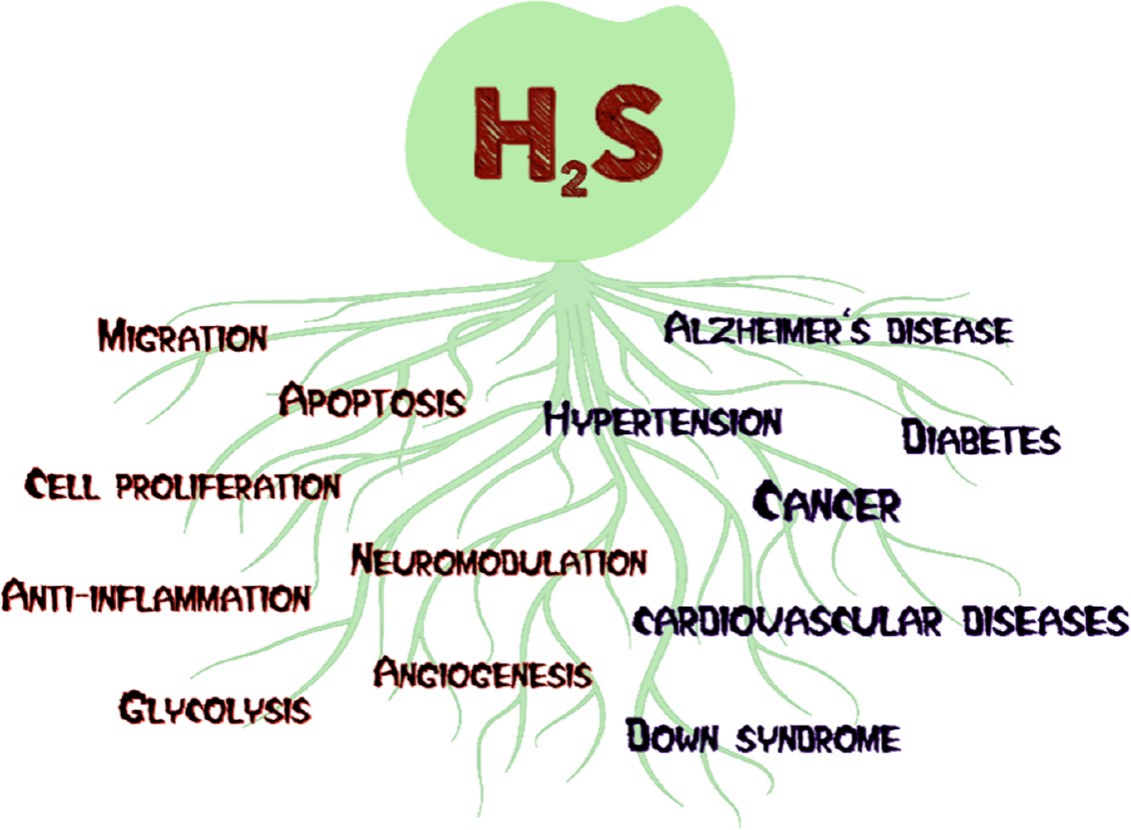
Illustration of H_2_S-involving pathological conditions and biological processes.

H_2_S is involved in a diverse range of biological processes, including glycolysis,^20,21^ anti-inflammation,^22−24^ migration,^25,26^ cell proliferation,^27−29^ neuromodulation,^30−32^ apoptosis,^33,34^ and angiogenesis^35,36^ among others. In addition, it is associated with various pathological conditions, such as diabetes,^37−39^ Alzheimer’s disease,^40−43^ Down syndrome,^44,45^ cardiovascular diseases,^46,47^ hypertension,^46−50^ and cancer^51−53^ (Figure 1). H_2_S is overexpressed in different types of cancer cells due to the elevated expression of enzymes responsible for its production in these cells.^51^ Therefore, it is essential to monitor and characterize the role of H_2_S in biological activities, and it can also be utilized as a key initiator for therapeutic action.

### 1.2 Phototherapy

**Figure 2:**
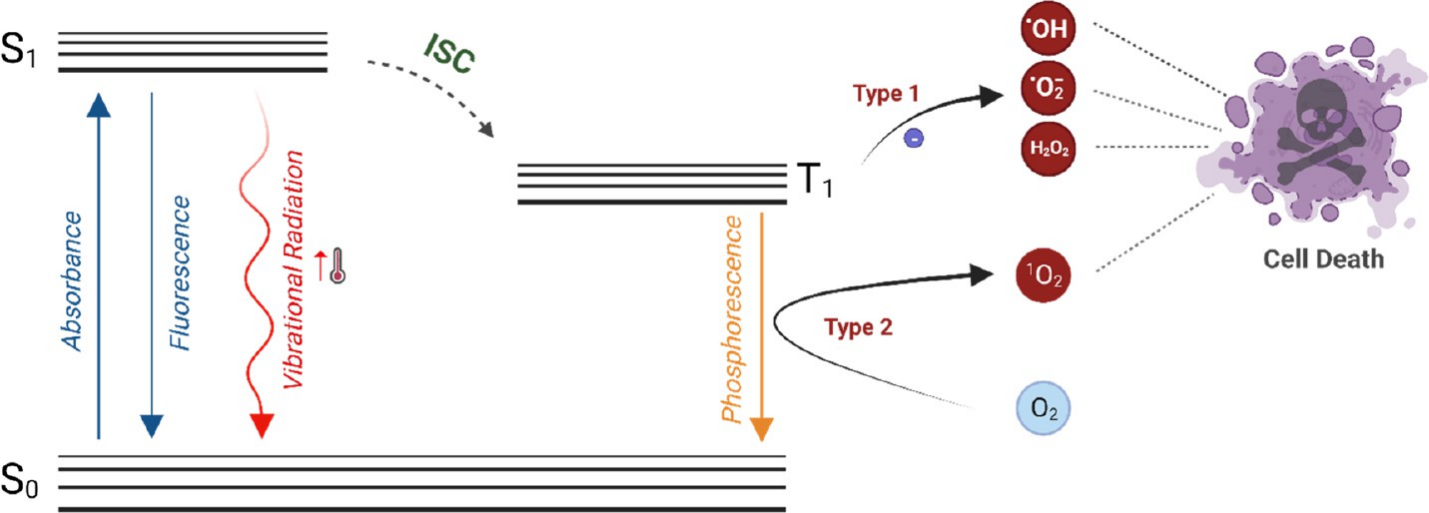
Schematic illustration of a modified Jablonski diagram.

Photodynamic therapy (PDT) is a clinically approved, noninvasive treatment modality used for the treatment of cancer and a range of nonmalignant diseases by employing cytotoxic reactive oxygen species (ROS).^54−61^ PDT holds unique advantages compared to conventional therapies including minimal side effects, no drug resistance, and activation of the immune system.^57,61−64^ Typical PDT involves three key elements: an outer light source, a photosensitizer, and tissue oxygen. In the presence of light, the photosensitizer (PS) is transferred from the S_0_ ground state to the S_1_ singlet excited state. The excited PS then may follow the S_1_ to S_0_ relaxation pathway while emitting light known as fluorescence, or it may follow nonradiative relaxation, which releases heat. Furthermore, it may undergo intersystem crossing (ISC) and switch to the first triplet excited state, T_1_. At that point, a photosensitizer either generates free radicals as a result of electron or proton transfer from T_1_ to biological substrates, favoring type I PDT, or transfers its energy to tissue oxygen (^3^O_2_), generating ^1^O_2_ through a type II mechanism. In addition, it may return to the ground state from the T_1_ excited state through phosphorescence (Figure 2). The majority of current PSs follow the type II pathway, and accordingly singlet oxygen is the major cytotoxic agent. It is worth mentioning that there is a growing interest in type I PSs as they can function effectively under hypoxic conditions, which is a difficult condition for ^1^O_2_ generating type II PSs.^57,65,66^ Photothermal therapy (PTT), on the other side, induces local temperature increase upon irradiation of PSs.^67−70^ PTT does not consume tissue oxygen; thus, it is highly attractive for the treatment of hypoxic tumors as in the case of type I PDT. In the case of both PDT and PTT, cancer cell selectivity is still highly sought to fully eliminate the adverse effects on healthy cells. Phototherapies are known to have intrinsic selectivity, as the irradiation light can be directed to the lesion area. However, in most cases this type of selectivity is not sufficient, and more sophisticated PS designs are needed. To this end, new generation PSs that can be activated solely in cancer cells are highly attractive. These activity-based PSs stay in their OFF state in nonmalignant cells even under light irradiation and turn on their cytotoxicity after getting activated in cancerous cells with a tumor-associated input such as biothiols, enzymes, and reactive oxygen species. H_2_S is among the most attractive analytes and has been utilized in numerous designs. The activity-based approach is also followed in the design of molecular sensors (activity-based sensors, ABS) to detect and image analytes of interest selectively. In such designs, the activity and concentration of the target analyte are strongly correlated to the response of activity-based probes. On the other hand, traditional binding-based designs exhibit an output signal upon binding or interaction with a specific target. Although they report on the concentration of the target, such as an enzyme, they do not provide information about their activity as opposed to activity-based agents.^71−73^

Activity-based phototherapy agents propose a notable success in cancer targeting, as they mostly remain in their off-state in normal cells. However, it is also possible that selected analytes can be expressed in different regions of the body or the serum itself. H_2_S, specifically, is involved in various physiological processes as explained in detail. Therefore, activation of the probe in different regions might damage vital organs in the body upon light exposure. One possible solution to address this issue is promoting activation of the agents within the target lesion by intratumoral or peritumoral injection. However, the ultimate goal of the new generation of photosensitizers is to trigger activation in the targeted zone, even after intravenous injection. Therefore, additional approaches have been employed to enhance the targeting and selectivity. One option in this direction is to utilize cell surface receptors (e.g., biotin, folate, human epidermal growth factor, etc.) that are differentially expressed in cancer cells.^74−78^ To this end, PSs are modified with specific groups that can bind to the receptor of interest selectively. Tumor-homing peptides, which are capable of penetrating the cell membrane, recognizing tumor-related antigens or proteins, and binding to specific cell surfaces, are highly popular. The most well-known tumor-homing peptide, RGD, is composed of a tripeptide chain (Arg-Gly-Asp) and binds to integrins α_V_β_3_ and α_V_β_5_.^74,79,80^ NGR is also composed of a tripeptide chain (Asp-Gly-Arg) and exhibits specific selectivity toward aminopeptidase N.^74,81^ Chlorotoxins, a small group of peptide toxins derived from scorpion venom, have been employed as targeting units in various studies, but they are particularly used for glioblastoma.^74,80^ In addition to the peptide family, glucose metabolism can be targeted via glucose transporters.^75,77,82^ Proteins such as transferrin and apolipoproteins have also been employed as targeting moieties.^74,83,84^ Furthermore, a range of vitamins have been utilized as cancer targeting ligands.^75^

In recent years, the combination of fluorescence imaging and phototherapy, in the form PDT, PTT, or multimodal PDT + PTT, on a single PS has emerged as a striking approach. These theranostic PSs enable the selective treatment and imaging of cancer cells at the same time. There are several reviews covering recent advancements in the field of activatable photosensitizers and multimodal phototheranostic agents.^54,55,57,60^ Fluorescent probes that aim to detect biothiols have also been summarized in several review papers.^85−87^ Herein, the scope of this review is to introduce current H_2_S responsive phototherapy and phototheranostic agents and their applications in both *in vitro* and *in vivo* models.

### 1.3 General Mechanisms for H_2_S-Induced Activation

In the design of H_2_S-responsive phototherapeutic agents, the common approach is to mask the PS core with a H_2_S-cleavable caging unit to quench the photo-cytotoxicity of the PS. Selective removal of the cage group then activates the therapeutic action by modulating the photophysical properties of the PSs. This modulation can be satisfied through different mechanisms including photoinduced electron transfer (PeT), intramolecular charge transfer (ICT), aggregation-induced emission (AIE), and energy transfer processes.^88^ Selective removal of the masking units and activation of the probes with H_2_S are commonly based on nucleophilic reactions, reduction, and sulfidation. Reduction-based activation mostly targets azide, nitro, nitroso, or azo groups. For instance, H_2_S-mediated azide to amine reduction leads to 1,6-elimination, which subsequently results in the release of the active scaffold while activating the ICT process.^89^ High nucleophilicity of H_2_S is mostly utilized to trigger nucleophilic aromatic substitution, disulfide bond cleavage, or Michael addition reactions.^90−92^ Nitrobenzene rings, for example, generally tend to undergo nucleophilic aromatic substitution, leading to the release of active phototherapeutic agents, which possess altered photophysical properties due to the modulation of PeT and/or ICT processes. 7-Nitro-1,2,3-benzoxadiazole (NBD) is another well-known group that is cleaved upon nucleophilic attack of H_2_S.^93^ Sulfidation is mostly satisfied as a result of reaction taking place between H_2_S and Cu^2+^ in which copper sulfide (CuS) is precipitated.^94^ Removal of Cu^2+^ by H_2_S changes the photophysical properties of the agents. Each of these mechanisms is discussed in detail through specific examples in the following sections.

## 2 H_2_S-Responsive Photodynamic Therapy (PDT) Agents

**Figure 3:**
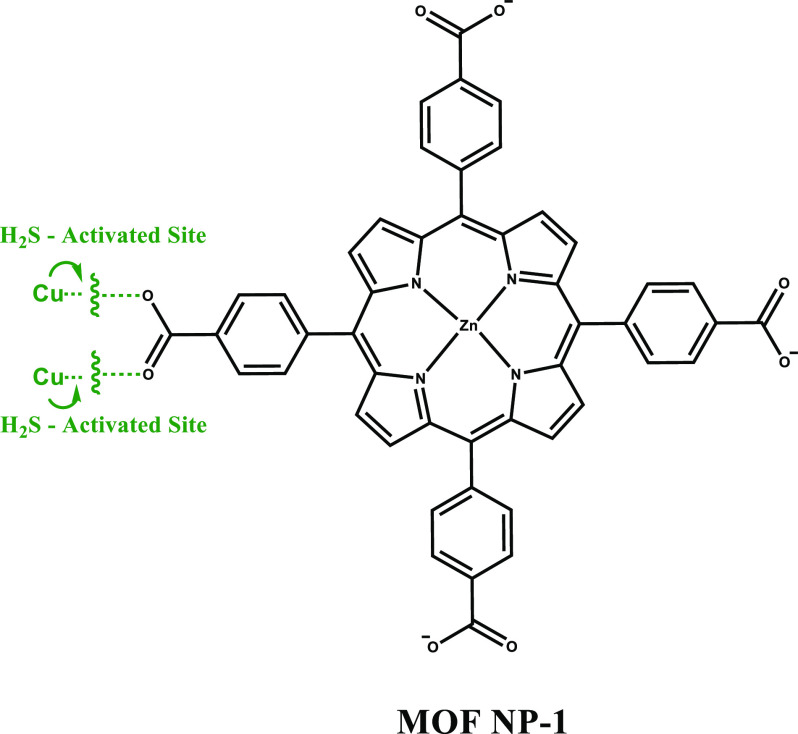
Reaction mode of MOF NP-1 with H_2_S and its chemical structure.

Considering the relationship between H_2_S and tumorigenesis, a variety of H_2_S activatable phototherapy agents have been developed. In this section, H_2_S-responsive PDT agents are introduced. In 2017, Ma et al. developed a novel photosensitizer that was extensively based on metal–organic framework nanoparticles (MOF NPs) in which the advantages of both nanostructures and the inherent features of crystalline MOFs were combined.^95^ The metal nodes of this network were constructed using Cu^2+^ ions, as these paramagnetic ions would interact with H_2_S and activate, otherwise quenching the emission of the MOF NP molecules (Figure 3).

**Figure 4:**
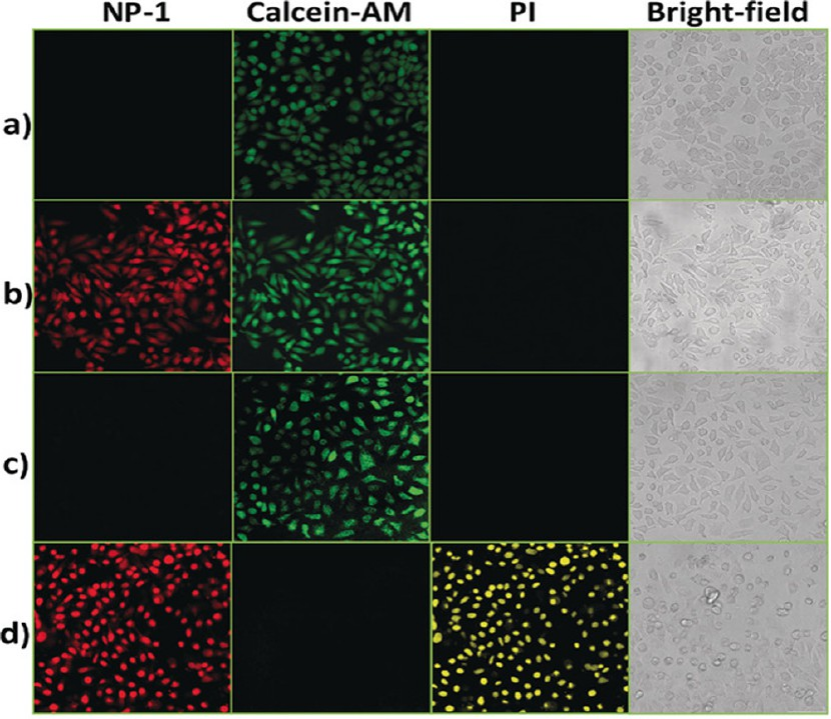
HepG2 cells treated with calcein-AM and PI in the presence of NP-1 (10 μM). (a) In the absence of light. (b) Exogenous treatment with NaHS (50 μM) in the absence of light. (c) Under light exposure. (d) Exogenous treatment with NaHS (50 μM) under light exposure. All images were captured by confocal microscopy. Adapted with permission from ref (95). Copyright 2017 Wiley-VCH.

This nanoscale copper–zinc MOF NP (NP-1) was prepared via a hydrothermal microemulsion with the formula of {Cu_2_(ZnTcpp)·H_2_O}. After HS^–^ treatment, the emission signal of NP-1 was increased, in a concentration dependent manner, around 610 and 660 nm with two shoulder peaks upon excitation at 420 nm. NP-1 was generating controllable ^1^O_2_ as it is only activated in the presence of H_2_S while its generation was negligible in the absence of H_2_S. Selective activation of NP-1 was also confirmed in HepG2 cells (human hepatocellular liver carcinoma cells) under confocal microscopy using propidium iodide (PI) and calcein-AM. Exogenous treatment with NaHS activated the probe, and strong red fluorescence was recorded on the red channel while the calcein-AM channel remained blank. As NP-1 stayed in its “on” state, singlet oxygen generation was initiated upon light exposure, which eventually caused cell death. As a result, a strong fluorescence intensity was obtained from PI (Figure 4). Additionally, the *in vivo* antitumor efficacy of NP-1 was evaluated on cancer cells having different levels of H_2_S expression. Tumor growth was not inhibited in HepG2 and LoVo (human colorectal adenocarcinoma cell) cells, which exhibit low levels of H_2_S. However, the tumor growth was suppressed successfully in the H_2_S overexpressing HCT116 cells.

**Figure 5:**
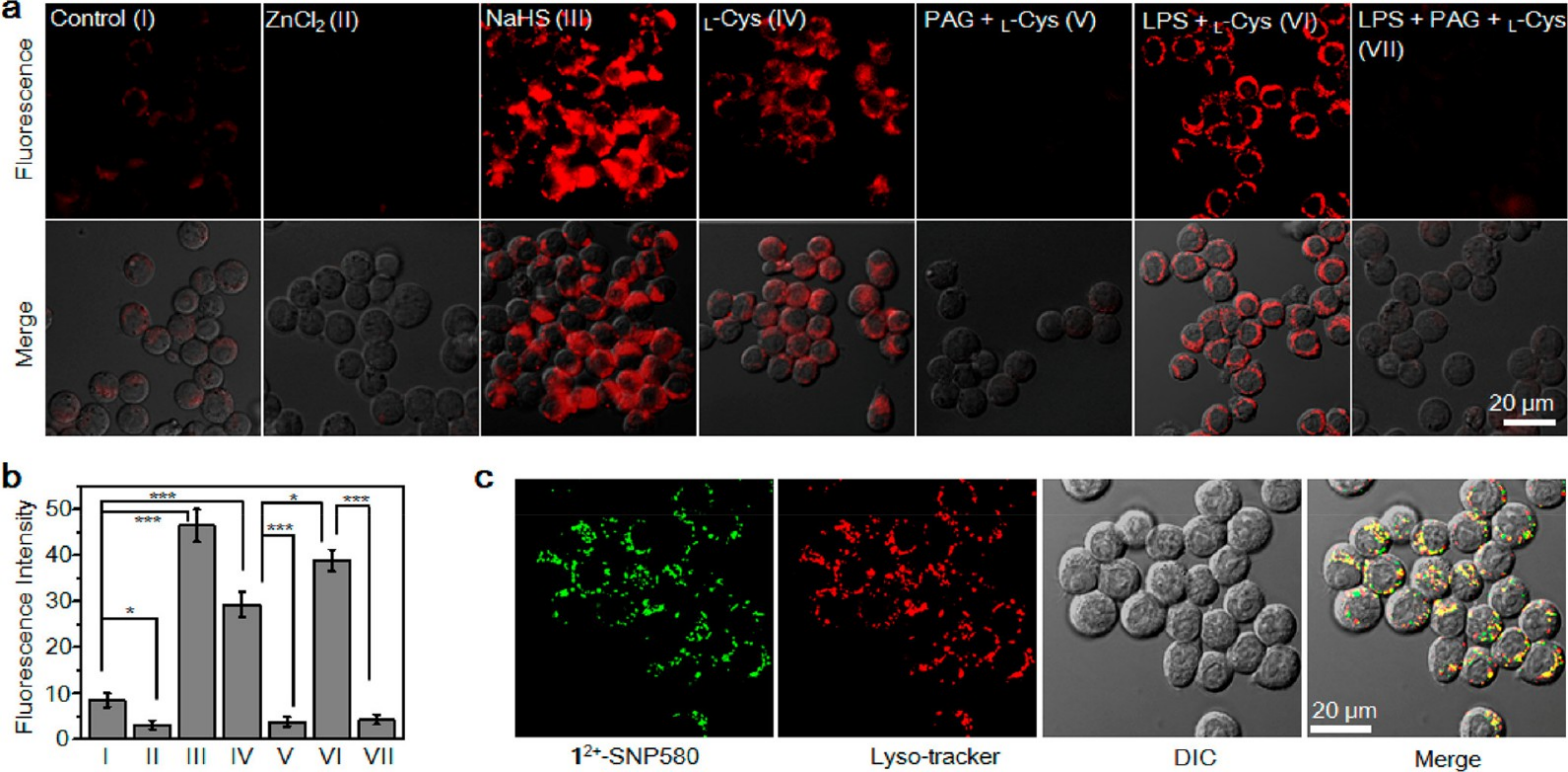
(a) Fluorescence images of RAW264.7 macrophages treated with the probe in the presence of enhancer, inducer, or inhibitor. (b) Corresponding fluorescence intensities. (c) Co-localization of **1^2+^**-SNP580 with Lyso-tracker red. Adapted with permission from ref (96). Copyright 2018 American Chemical Society.

Following the previous study, Wu et al. engineered electrochromic H_2_S activatable probes for imaging and PDT.^96^ The authors acknowledged the previous work but stated that MOF NPs lack selective targeting of cancer cells and NIR light absorption, which restricts PDT action *in vivo*. 1,1,4,4-Tetra-aryl butadiene (EM **1**^**2+**^), an organic π-electron structure with a sharp absorption band at 500 and 850 nm and H_2_S responsive characteristic, was modified to get activatable fluorescence probes (**1^2+^**-SNP580, **1^2+^**-SNP700, and **1^2+^**-SNP830) and PSs. EM **1^2+^** doped with semiconducting polymers initially showed no fluorescence due to fluorescence resonance energy transfer (FRET), but its emission was recovered once reduced to diene EM **2** in the presence of H_2_S. All the fluorescence probes selectively and sensitively responded to H_2_S and the fluorescence response of **1^2+^**-SNP830 fluctuated in accordance with the treating enhancer, inducer, or inhibitor (Figure 5). **1^2+^**-SNP830 and **1^2+^**-SNP580 responded to endogenous H_2_S in RAW264.7 macrophages and were primarily localized on lysosomes (Figure 5). Besides, the one working in the near-infrared region, **1^2+^**-SNP830, accurately measured H_2_S content in human plasma. **1^2+^**-SNP830 also monitored hepatic H_2_S activity *in vivo*. Folic acid was employed to enhance targeting abilities of **1^2+^**-SNP830 along with the DSPE-PEG2000/DSPE-PEG2000-FA extension. Once **1^2+^**-SNP830-FA was prepared using the nanoprecipitation method, it monitored H_2_S-rich HT29 and HCT116 colorectal cancer cells efficiently together with H_2_S content in KB tumor-bearing mice. As a final approach, R6G and NIR775, whose absorption signals were similar to those of 1^2+^_,_ were selected as controllable ^1^O_2_ generating scaffolds to prepare **1^2+^**-PSs-FA. A sensitive turn-on response was detected for H_2_S with a very low limit of detection (LOD) value, approximately 19 and 39 nM at 555 and 780 nm, respectively. As shown by experiments with singlet oxygen sensor green (SOSG), in the presence of NaHS, **1^2+^**-PSs-FA was activated, and its ^1^O_2_ generation was initiated upon 808 nm laser irradiation, while negligible response was observed from the sensor in the absence of NaHS. *In vivo* studies have shown that intravenously injected **1**^**2+**^-PSs-FA was localized primarily to tumors and activated with endogenous H_2_S. After laser irradiation at 808 nm, tumor growth was inhibited notably because of effective PDT action, while no toxicity was observed in major organs. Furthermore, once tumors were stimulated by exogenous l-Cys, a vital substrate in the H_2_S production pathway, the PDT efficacy was enhanced.

**Figure 6:**
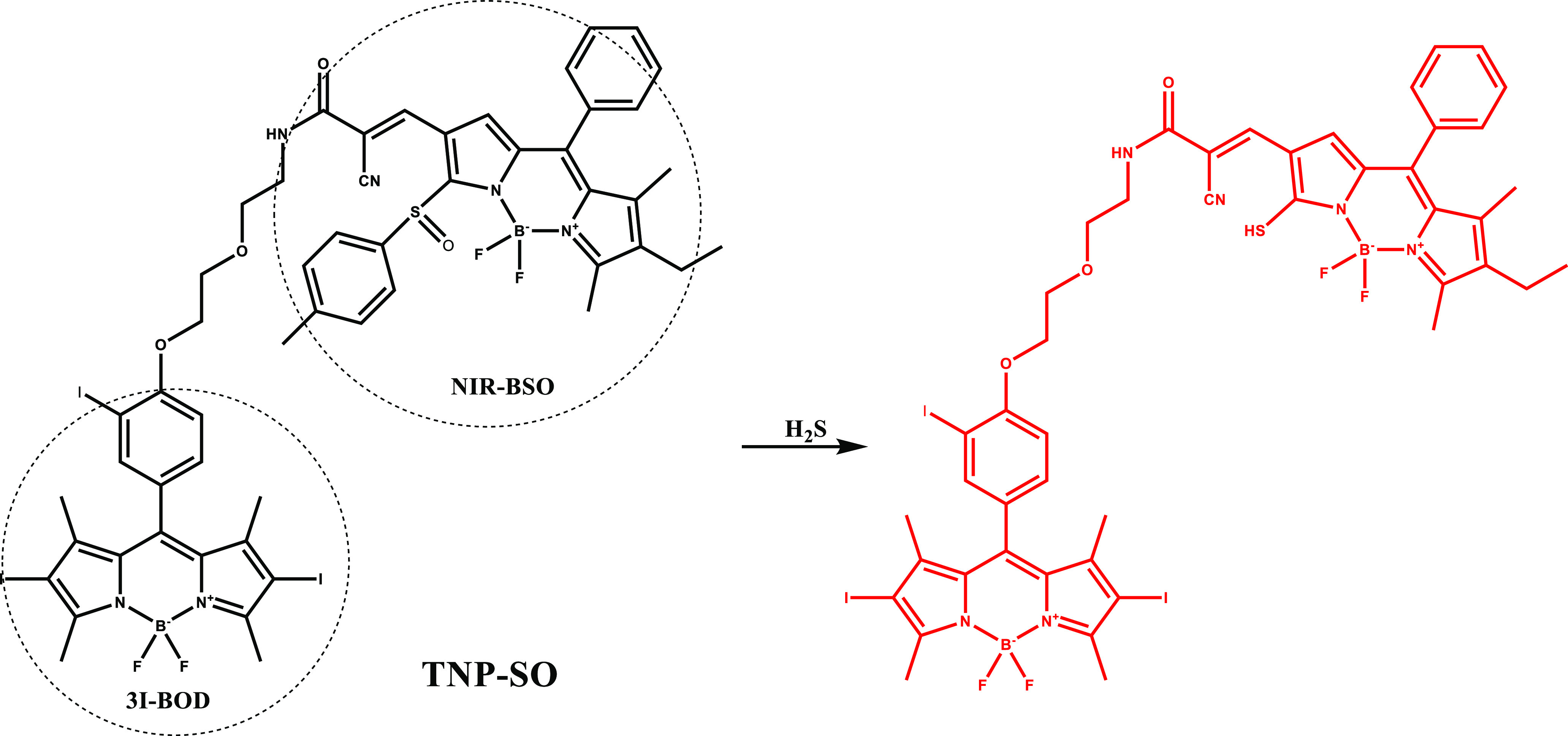
Structure and activation of TNP-SO with H_2_S.

In 2019, Wang et al. proposed a theranostic prodrug platform (TNP-SO), a combination of a H_2_S-responsive imaging agent (NIR-BSO) and a photosensitive drug (3I-BOD) (Figure 6).^97^ Due to low water solubility of TNP-SO, the core was encapsulated in water-dispersible silica nanocomposites, named as nano-TNP-SO. After H_2_S treatment, nano-TNP-SO exhibited a red-shift from 537 to 677 nm in the absorption signal resulting in 137-fold enhancement in the emission signal at 712 nm after irradiation at 640 nm. Nano-TNP-SO was activated selectively in H_2_S-rich HCT116 cells and exhibited light induced toxicity but remained inactive in HepG2 cells. Besides, nano-TNP-SO displayed minimal dark toxicity in HCT116 cells. Ten days after treatment, nano-TNP-SO inhibited tumor volume with 82% efficiency.

**Figure 7:**
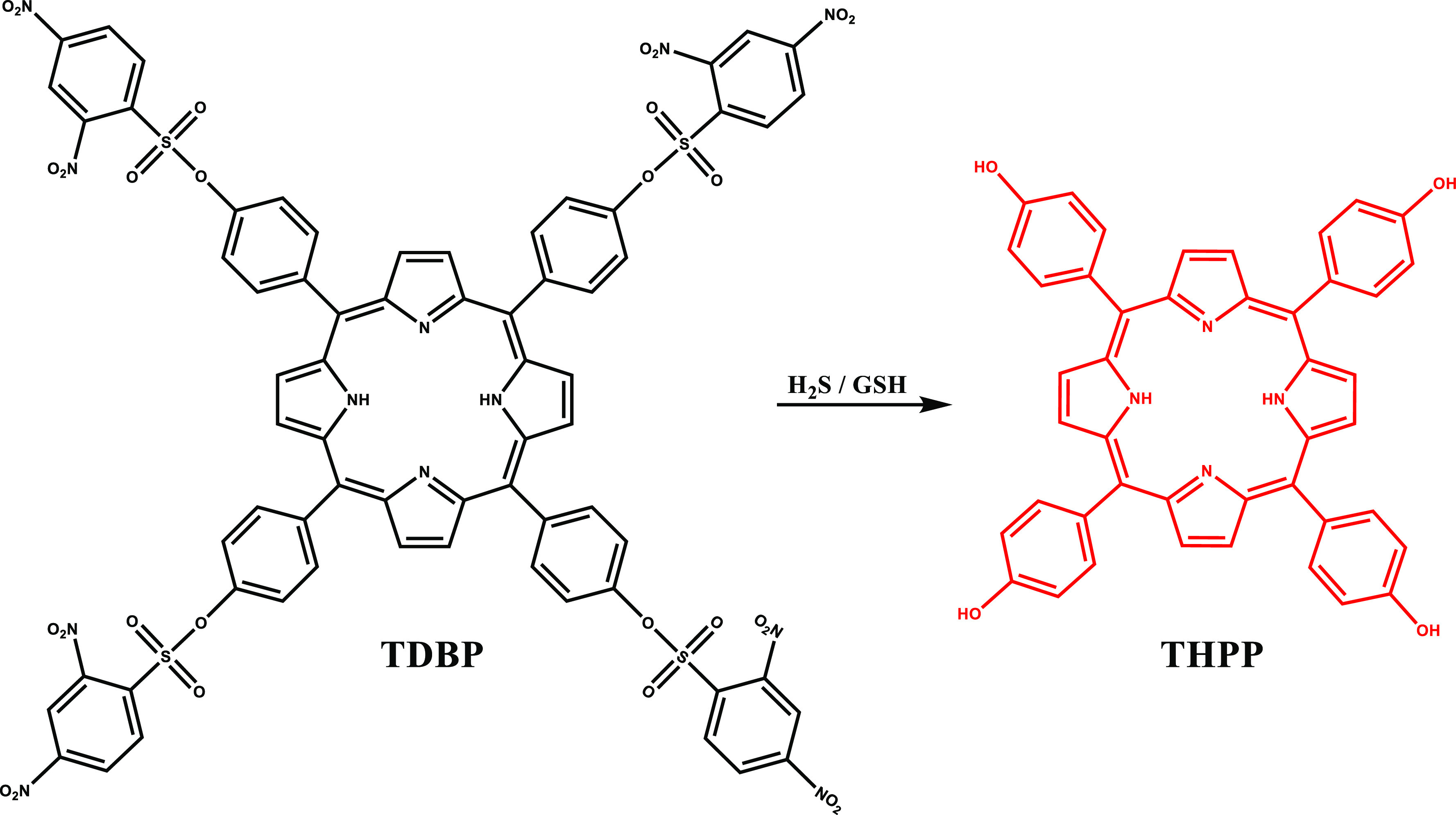
Structure and activation of TDBP with H_2_S.

A H_2_S/GSH dual responsive activatable PS (aPS), TDBP, was developed by Huang et al. in 2022.^98^ TDBP was composed of an active 5,10,15,20-tetra(4-hydroxyphenyl)porphyrin (THPP) core and a H_2_S/GSH responsive quencher (Figure 7). TDBP was activated in the presence of H_2_S or GSH, which restored its fluorescence at 660 nm, as well as its ROS generation ability. Upon activation by endogenous H_2_S or GSH, TDBP exhibited photocytotoxicity in HCT116 cells, but negligible cytotoxicity was observed in normal human hepatocyte LO2 cells under light irradiation or under dark conditions.

**Figure 8:**
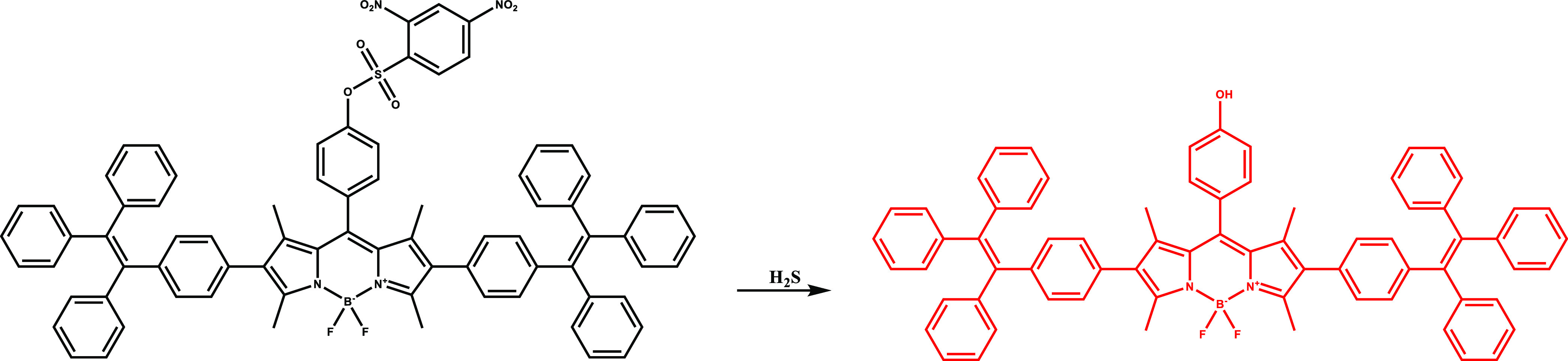
Structure and activation of DB2T with H_2_S.

In 2022, the BODIPY core was combined with tetraphenylethene (TPE) units and H_2_S sensitive moiety to construct H_2_S responsive PS (DB2T) by Quan et al. (Figure 8).^99^ DB2T responded to H_2_S selectively with low LOD (6.39 nM) and restored its fluorescence at 579 nm upon excitation at 534 nm.

**Figure 9:**
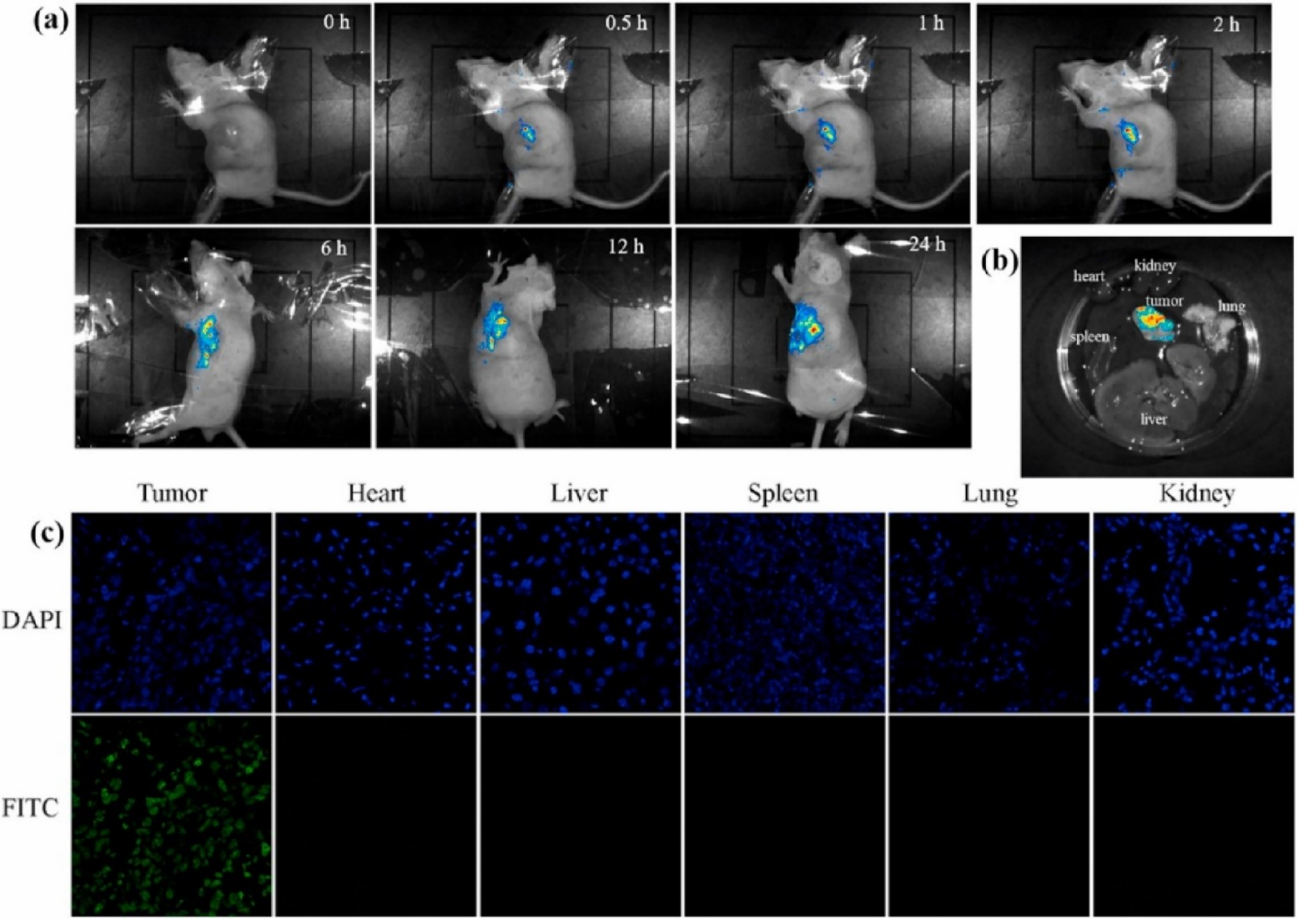
(a) Fluorescence images of HCT116-bearing mice recorded at different times after injection of DB2T. (b) Fluorescence images of vital organs recorded 24 h after DB2T injection. (c) DAPI and FITC treated slices of mice recorded under confocal microscopy 24 h after DB2T injection. Adapted with permission from ref (99). Copyright 2022 Elsevier.

*In vitro* studies revealed that DB2T displayed selective activation in cells as it was activated in HCT116 cells but remained nonfluorescent in HepG2, PC12, or HUH-7D cells. Additionally, DB2T exhibited photo-cytotoxicity in HCT116 because of efficient ROS generation. DB2T switched on its fluorescence selectively with endogenous H_2_S and successfully monitored the tumor region in HCT116-tumor-bearing living nude mice (Figure 9). In contrast, DB2T was activated only in the presence of exogenous H_2_S in zebrafish.

**Figure 10:**
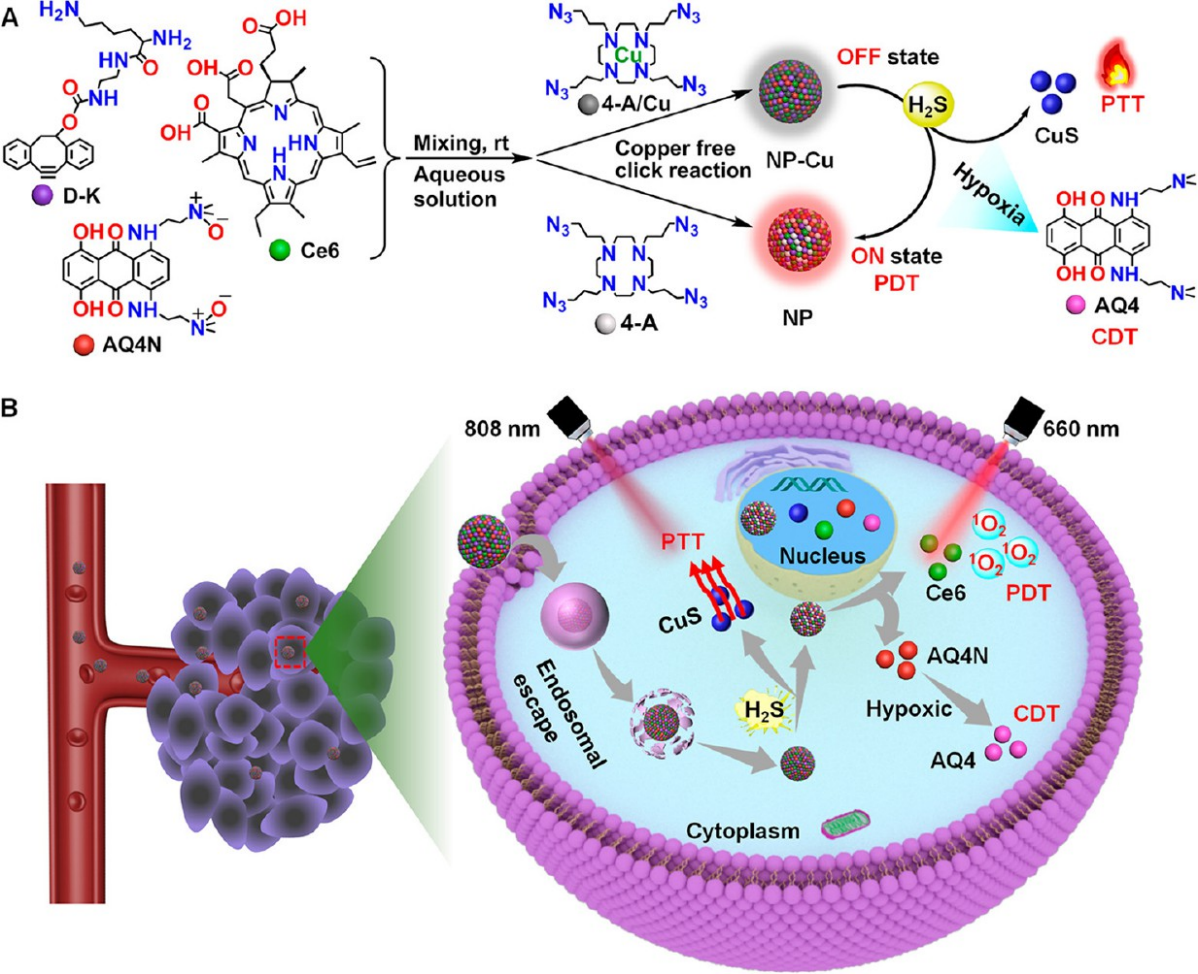
(A) Schematic depiction of NP-Cu preparation and (B) its activation with endogenously produced H_2_S. Adapted with permission from ref (100). Copyright 2022 American Chemical Society.

An example of a nanoplatform offering trimodal synergistic therapy, PDT, PTT, and chemodynamic therapy (CDT), was devised by Yang et al. in 2022.^100^ The nanocomplex NP-Cu was fabricated using dibenzocyclooctyne (DBCO) functionalized lysine (D-K) and an azamacrocylic ring (4A/Cu) along with a photosensitizer, chlorin e6 (Ce6), and hypoxia-activated prodrug banoxantrone (AQ4N) (Figure 10). As Cu^2+^ quenched the emission of Ce6, initially the NP-Cu remained in its off state, but introduction of H_2_S turned PDT to its on-state, and in harmony, endogenous H_2_S reacting with Cu^2+^ generated CuS particles, a potent PTT agent. Synergistically, consumption of oxygen due to PDT promoted hypoxia and activated AQ4N, which resulted in CDT and eventual trimodal therapy (Figure 10). *In vitro* studies indicated that NP-Cu was activated by endogenous H_2_S in HCT116 cells selectively, turned on therapeutic channels of trimodal therapy, and displayed selective toxicity. In accordance with the *in vitro* results, NP-Cu displayed high antitumor activity due to a trimodal cascade therapy.

A mitochondria-targeted aggregation-induced emission (AIE) PS, which favors type I PDT, was reported by Zhang et al. in 2022.^101^ The type I AIE PS (TDCAc) was encapsulated with a H_2_S donor, (NH_4_)_2_S, and then further constructed on a hydrogel system (TSH). Temperature was elevated upon light irradiation at 660 nm and released previously encapsulated TDCAc aggregates along with (NH_4_)_2_S. While TDCAc aggregates localized at mitochondria due to ionic interactions, H_2_S was generated from (NH_4_)_2_S in an acidic tumor microenvironment. This inhibited catalase activity, which resulted in continuous radical formation, with the assistance of a labile iron pool, due to TDCAc induced H_2_O_2_ generation. The design approach followed here did not rely on H_2_S activation but proposed a way to utilize H_2_S in synergistic therapy for enhanced PDT efficacy.

**Figure 11:**
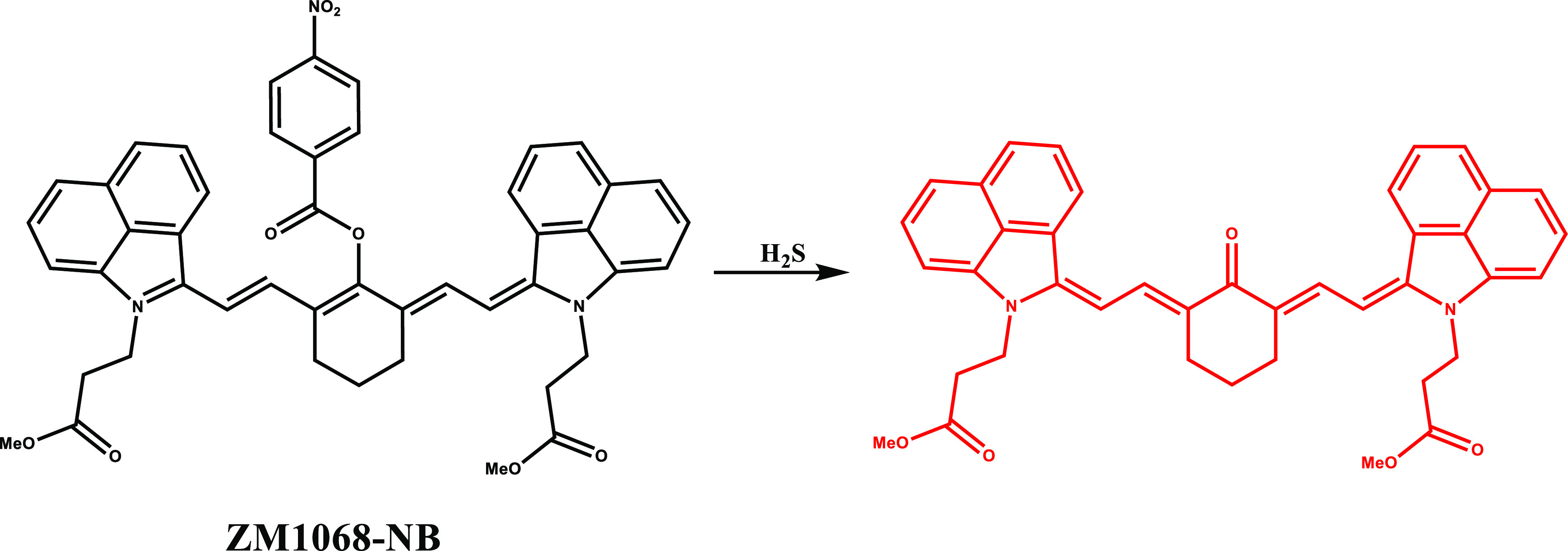
Structure and activation of ZM1068-NB with H_2_S.

In 2022, Zhang et al. presented a novel nanoplatform, ZNPPs, with H_2_S responsive and depleting characteristics for real-time monitoring of H_2_S activity in biological systems and delivering PDT action (Figure 11).^102^ ZM1068-NB was first synthesized by following a six-step synthetic route, then ZNNPs and ZNNPs@FA were prepared using a nanoprecipitation method with the assistance of mPEG_5000_-PCL_3000_ and mPEG_5000_-PCL_3000_-FA.

**Figure 12:**
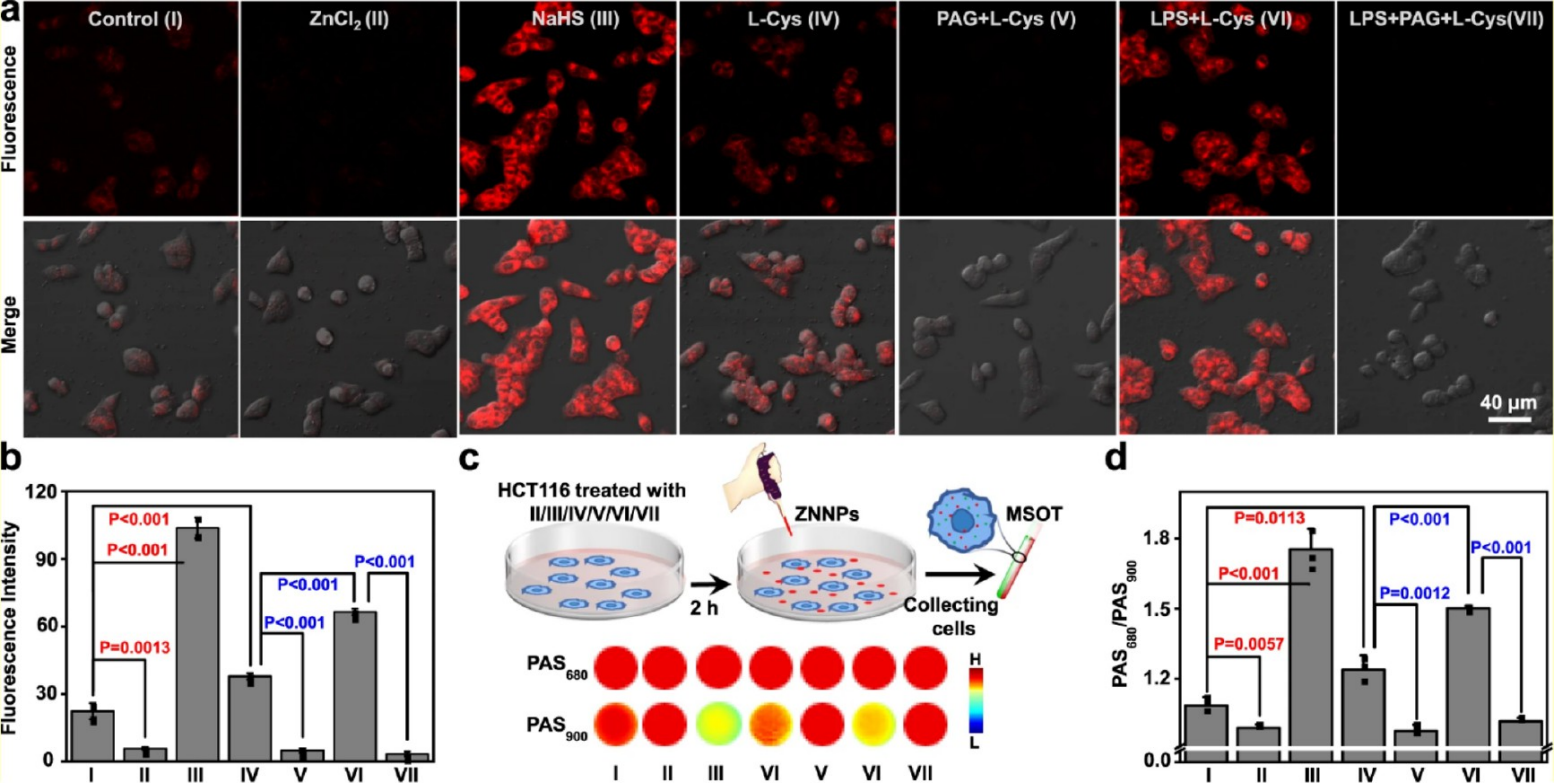
(a) Fluorescence images of HCT116 cells treated with the probe in the presence of enhancer, inducer, or inhibitor and (b) its corresponding fluorescence intensities. (c) PAS_680_ and PAS_900_ images and (d) corresponding PA signals (PAS_680_/PAS_900_). Adapted with permission under a Creative Commons CC BY License from ref (102). Copyright 2022 Springer Nature.

The active sites on these nanoprobes underwent substitution reactions with endogenous H_2_S and generated NIR shift in the fluorescence channel from 1070 to 720 nm along with a shift in the ratiometric photoacoustic signals from 900 to 680 nm. H_2_S introduction to ZM1068-NB resulted in a blue shift in the absorption maximum to 650 nm along with activated ^1^O_2_ generation upon 660 nm laser irradiation, which ultimately induced cell death. Photophysical characterization studies confirmed that ZNNPs not only responded to H_2_S selectively but also exhibited high linear correlation response within the range of 0 to 500 μM NaHS. ZNNPs were later successfully applied for the monitoring of endogenous H_2_S in HCT116 cells as well as photoacoustic imaging. Fluorescence signal was shown to decrease in HCT116 cells treated with ZnCl_2_ (40 μg/mL), a H_2_S quencher, or dl-propargyl glycine (50 μg/mL), a CSE inhibitor. In contrast, the fluorescence intensity increased upon treatment with l-Cys (24 μg/mL), an upregulator for H_2_S, or exogenous NaHS (1 mM) (Figure 12). Besides, the hepatic H_2_S level in mouse liver and endogenous H_2_S in the injured brain of mice was successfully evaluated using ZNNPs both through fluorescence and photoacoustic imaging. ZNNPs@FA inhibited tumor progression in HCT116 tumor-bearing BALB/c mice, and tumor size reduction up to 89.3% was reported. On the other hand, ZNNPs@FA was found to deplete intracellular H_2_S, thus preventing proliferation in HCT116 and contributing to PDT.

**Figure 13:**
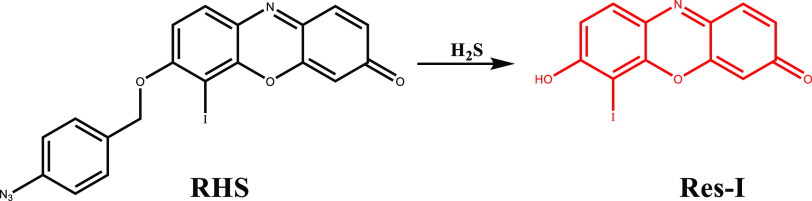
Structure and activation of RHS with H_2_S.

Recently, our group introduced an iodinated resorufin core-based H_2_S-responsive phototheranostic agent (RHS) for selective treatment and imaging of neuroblastoma.^103^ RHS itself was initially inactive as ICT was blocked. Selective removal of the masking unit on the hydroxyl group by H_2_S released the active Res-I core (Figure 13) and restored its emission at 606 nm upon excitation at 586 nm, while activating the PDT action.

**Figure 14:**
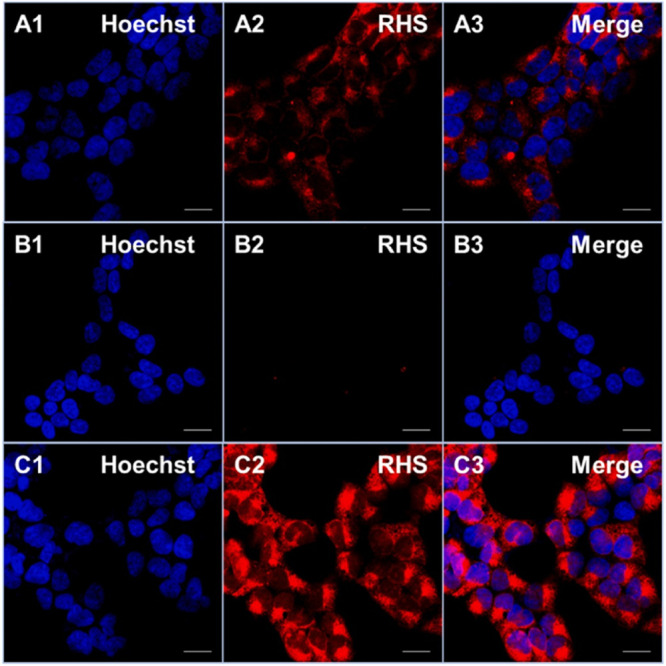
Confocal images of SH-SY5Y neuroblastoma cells. First row: cells were treated with RHS; Second row: cells were pretreated with NEM, and then incubated with RHS; Third row: cells were pretreated with NaHS and then incubated with RHS. The blue channel indicates HOECHST 33342 and the red channel indicates RHS. Adapted with permission from ref (103). Copyright 2023 Elsevier.

RHS possessed high singlet oxygen quantum yield (Φ_Δ_ = 0.42) and selective response to H_2_S, compared with other biologically relevant analytes. Selective activation of RHS in SH-SY5Y neuroblastoma cells was shown under confocal microscopy using *N*-ethylmaleimide (NEM) as a H_2_S inhibitor and NaHS as an inducer (Figure 14). RHS exhibited light induced cytotoxicity in H_2_S rich SH-SY5Y neuroblastoma cells (IC_50_ = 3.29 ± 0.18 μM) but remained inert in healthy L929 mouse fibroblast cells.

**Figure 15:**
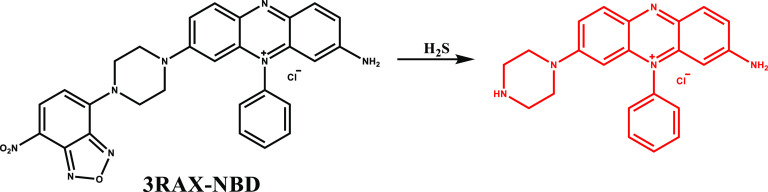
Structure and activation of 3RAX-NBD with H_2_S.

In 2023, Jia et al. incorporated a H_2_S sensitive competent (NBD) to phenazinium derived methylene violet 3RAX dye to construct a H_2_S depletion aided PDT platform (3RAX-NBD) (Figure 15).^93^ In the presence of H_2_S, the probe underwent a slight blue shift from 560 to 540 nm and resulted in amplified singlet oxygen generating ability along with enhanced fluorescence at 610 nm upon irradiation at 550 nm. 3RAX-NBD displayed no dark toxicity but photoinduced cytotoxicity in the concentration region of 0–50 μM in 4T1, HeLa, and MCF-7 cancer cells. PDT efficiency of the probe was later evaluated in 4T1 tumor-bearing BALB/c mice, and the tumor size was reduced by 91.3% under 550 nm laser irradiation, while no reduction in tumor size was noted in the control groups.

## 3 H_2_S-Responsive Photothermal Therapy (PTT) Agents

**Figure 16:**
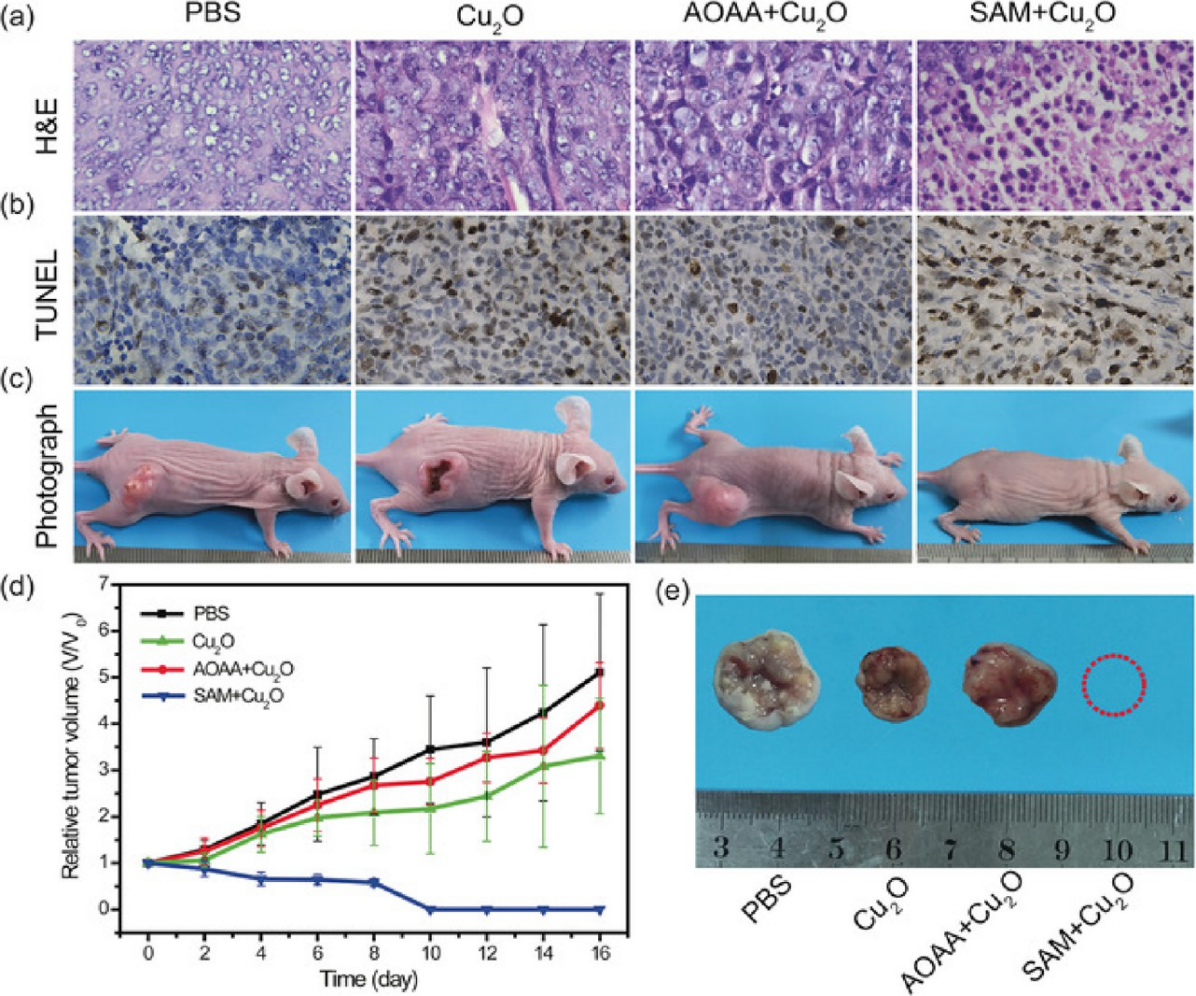
Histological assessment of (a) H&E and (b) TUNEL stained tumor regions. (c) Photographs of mouse models captured after 16 days. (d) Evaluation of tumor progression captured every 2 days. (e) Photographs of tumor regions captured after 16 days of treatment from different groups. Adapted with permission from ref (104). Copyright 2018 Wiley-VCH.

In 2018, Cu_2_O nanoparticles were prepared by An et al. for H_2_S activatable photoacoustic (PA) imaging and PTT.^104^ The oxide nanoparticles were converted to copper sulfide by H_2_S, enhancing PTT and activating PA. Under 808 nm laser irradiation, the sulfidized nanoparticles exhibited a concentration-dependent increase in the temperature and the PA signal. Photothermal conversion efficiency of the particles after NaHS treatment was determined to be 15.6%. Cu_2_O particles were selectively activated in HCT tumor-bearing mice, and tumor distribution was monitored through PA channels. Remarkably, the tumor completely disappeared on the 16th day as a result of PTT action in the S-adenosyl-l-methionine treated group; however, tumor volume increased gradually in positive and negative control groups (Figure 16).

**Figure 17:**
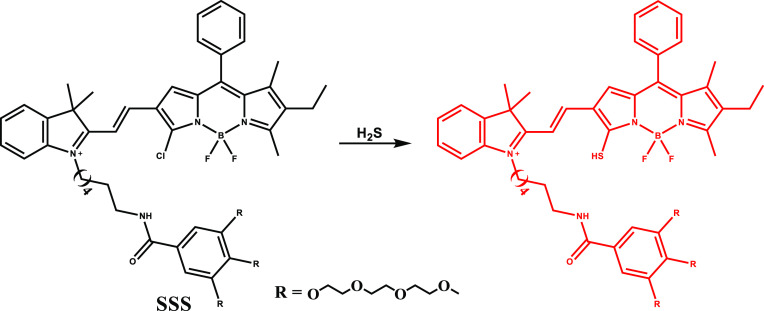
Structure and activation of SSS with H_2_S.

In 2018, Shi et al. constructed an example of a nanostructured NIR-II fluorescence-guided PTT agent with H_2_S responsive characteristics (nano-PT) using a monochlorinated BODIPY scaffold (SSS) that was extended with a hydrophilic tail in order to mediate self-assembly (Figure 17).^105^ Red shift in the absorption spectrum from 540 to 790 nm upon NaHS treatment enhanced photothermal conversion and increased the temperature of the solution containing nano-PT by 32 °C upon 785 nm NIR laser irradiation. Consistently, PTT was reported to rely on the concentration of the probe or analyte and the corresponding laser power. PTT efficacy of the nano-PT was proven successfully under confocal microscopy using calcein-AM and PI treated H_2_S rich HCT116 cells. Remarkably, *in vivo* studies indicated that after 10 min of NIR laser exposure, the temperature in the tumor region of HCT116 tumor-bearing mice rose to nearly 60 °C; as opposed to that, slight temperature change was observed in control groups.

**Figure 18:**
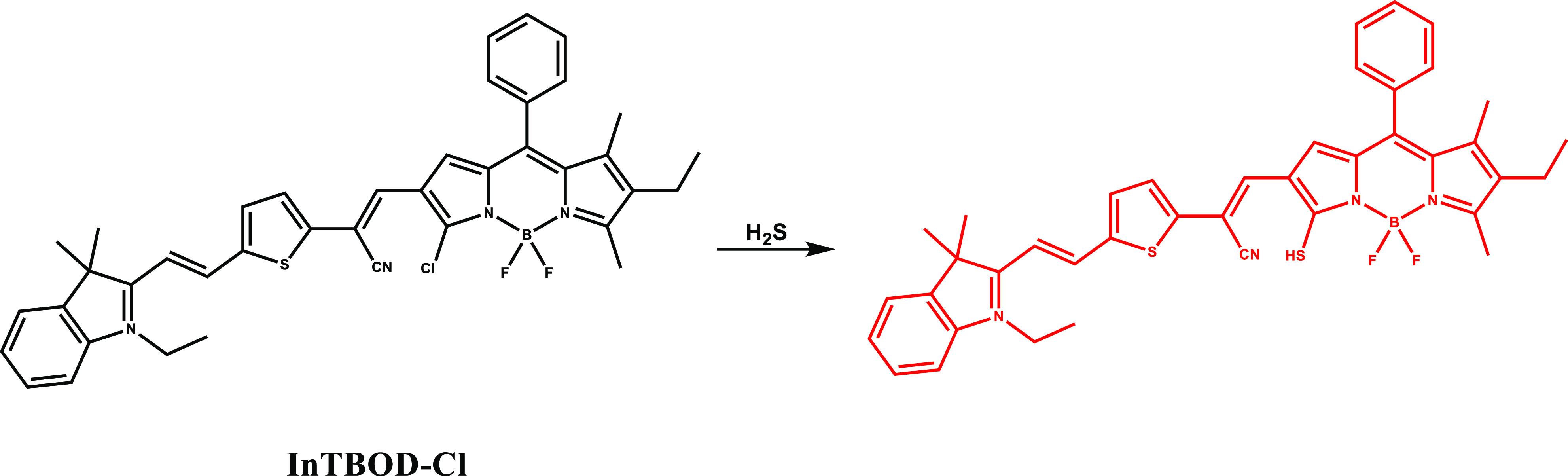
Structure and activation of InTBOD-Cl with H_2_S.

A nanoplatform (NPs@BOD/CPT) was established by the same group using a BODIPY derived (InTBOD-Cl) probe (Figure 18) for the aim of simultaneous combined therapy and imaging.^106^ The nanoplatform was assembled by coencapsulation of the BODIPY derived theranostic and camptothecin-11 (CPT-11), a chemotherapeutic drug. The thermal shift of the NPs@BOD/CPT under 785 nm laser exposure was modest in the absence of H_2_S, but introduction of H_2_S led to significant photothermal conversion and a concomitant temperature rise. At the eutectic melting point (39 °C), previously encapsulated CPT-11 was released and aided the therapy. In accordance with the design principle, NPs@BOD/CPT was turned on by H_2_S in HCT116 cells and released CPT-11 under NIR light irradiation. Similar on-demand photocontrolled drug release was observed in the *in vivo* studies. As a result, tumor growth was suppressed in HCT116 tumor-bearing mice after intratumoral injection of NPs@BOD/CPT and laser irradiation, but in dark conditions or in the absence of probe, no suppression was detected.

**Figure 19:**
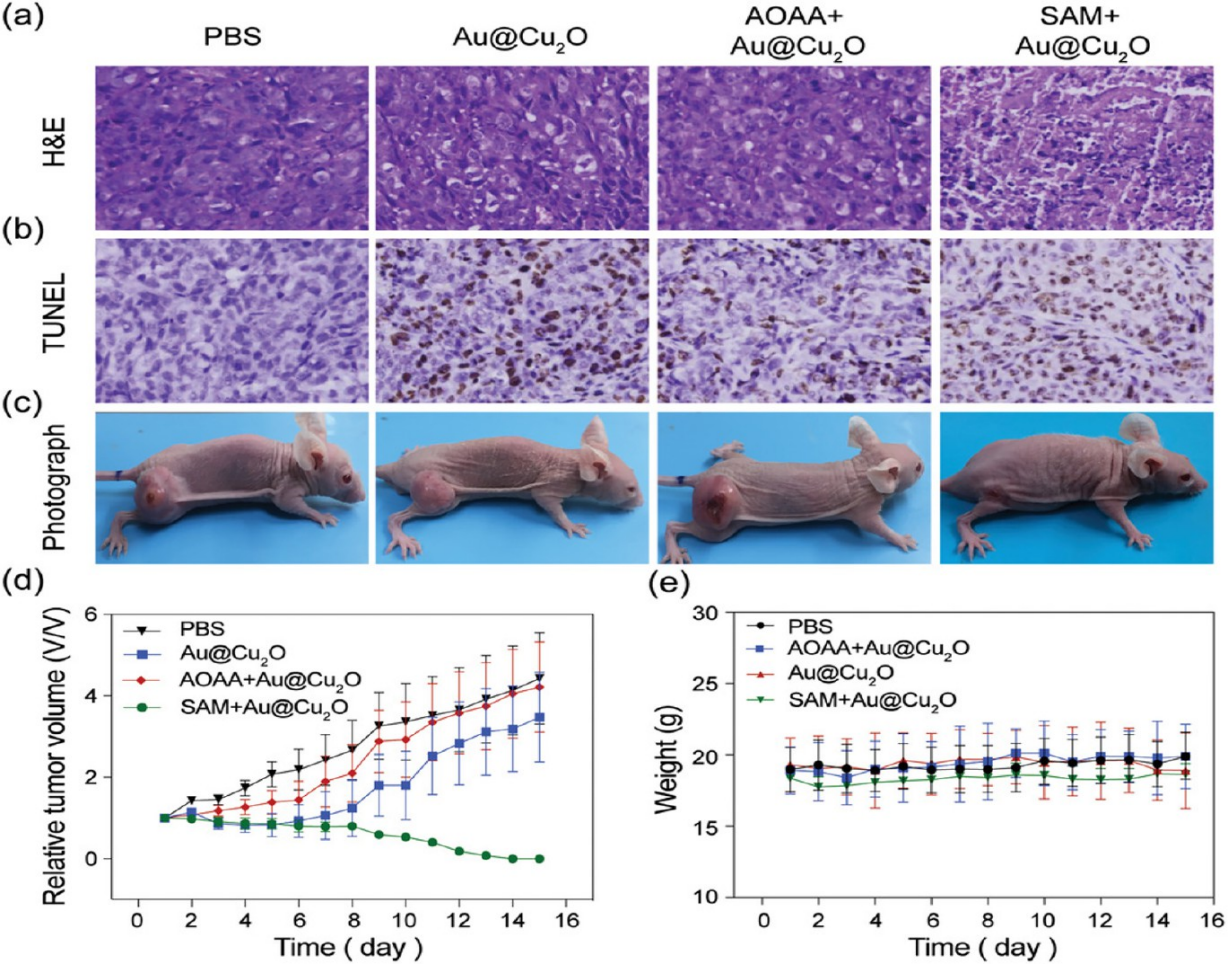
Histological analysis of (a) H&E and (b) TUNEL stained tumor regions. (c) Photographs of mouse models captured after 16 days (d) Evaluation of the tumor progression captured every 2 days. (e) Weight of the mice recorded during the course of therapeutic action. Adapted with permission from ref (107). Copyright 2019 Wiley-VCH.

In 2019, the probe Au@Cu_2_O was proposed by Tao et al. to realize PTT and PA imaging of H_2_S-rich cancer cells, where the localized surface plasmon resonance (LSPR) coupling effect between the Au nanosphere seed and the sulfidized Cu_2_O layer (Cu_9_S_8_) would enhance the photoacoustic contrast and promote PTT.^107^ The synthesis was carried out by coating the Cu_2_O layer on Au nanosphere seeds while using polyvinylpyrrolidone (PVP) as a surfactant. During kinetic studies, the Au@Cu_2_O system was reported to have an absorption maximum at 808 nm that displayed an increased intensity even at low concentrations of NaHS (0.08 mM), while providing an improved photoacoustic contrast change. Similarly, upon irradiation, Au@Cu_2_O was reported to cause dramatic change in temperature even with a low concentration of NaHS (0.014 mM). The *in vivo* experiments performed with HCT-bearing mice revealed that the groups treated with Au@Cu_2_O and S-adenosyl-l-methionine (SAM) + Au@Cu_2_O displayed high photoacoustic contrast when compared with the control groups, where the PA contrast of the SAM + Au@Cu_2_O group was reported to have surpassed that of the Au@Cu_2_O-only mice. Likewise, during the photothermal therapy trials, the tumor tissue was reported to have been eradicated in SAM + Au@Cu_2_O mice, while a minor change in the relative tumor volume was observed in the Au@Cu_2_O-only group over the course of 16 days (Figure 19).

**Figure 20:**
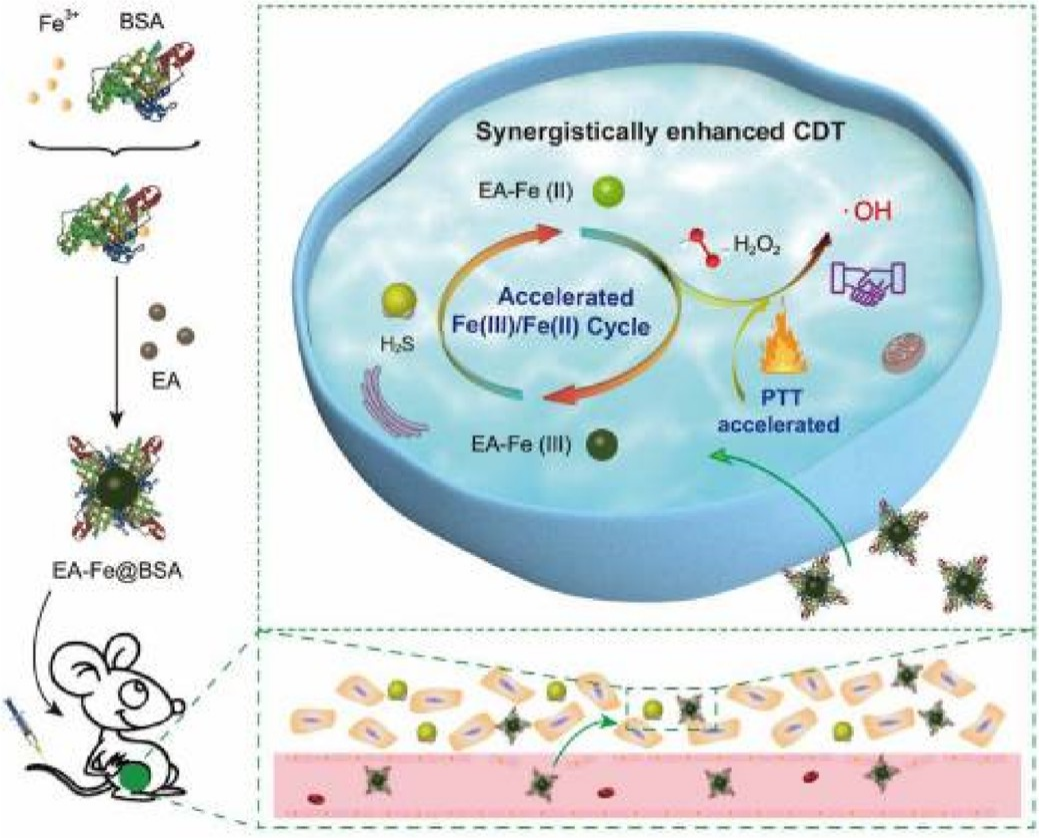
Schematic depiction of the construction of EA-Fe@BSA and illustration of accelerated conversion of Fe(III)/Fe(II) with H_2_S and synergistic therapy. Adapted with permission under a Creative Commons CC BY License from ref (108). Copyright 2020 Ivyspring International Publisher.

In 2020, a PTT-enhanced CDT probe (EA-Fe@BSA) was devised by Tian et al. to hasten the H_2_S-mediated Fe(III)/Fe(II) cycle, which enhances the yields of ROS that are generated via Fenton and Fenton-like reactions while synergistically improving the CDT efficacy via PTT (Figure 20).^108^ The synthesis of these nanoparticles was carried out by mixing an aqueous solution of FeCl_3_ with bovine serum albumin (BSA), followed by the addition of ellagic acid at room temperature. The *in vitro* studies carried out with HCT116 cells revealed that the NPs were able to promote the synthesis of hydroxyl radicals to great extents in the presence of NaHS and H_2_O_2_. The ROS-generating capacity of NPs was also seen to be improved with moderate heating. Upon irradiation of EA-Fe@BSA with an 808 nm laser, a concentration-dependent photothermal effect was detected with a conversion rate of approximately 31–32%. Additionally, the longitudinal (T_1_) and transverse (T_2_) relaxation times of the EA-Fe@BSA system proved it to be a viable T_1_-weighted MRI contrast agent that can provide concentration-dependent imaging of cells. The *in vivo* trials conducted with HCT116 mice were able to display the localization of NPs through MRI and their therapeutic effects. Total tumor ablation was achieved in the NPs + S-adenosyl-l-methionine (SAM) + laser group, while considerable tumor shrinkage was observed in the NPs + laser group. However, the laser-only, NPs-only, and NPs-SAM groups were not able to obtain such decreases in relative tumor volumes, implying the necessity of CDT/PTT combined therapeutic approaches that are linearly dependent on the concentrations of H_2_S.

A multifunctional cascade activated theranostic nanosystem (AB-DS@BSA-N_3_) bearing diallyl trisulfide (DATS), a H_2_S donor, and a H_2_S sensitive azide functional group was synthesized by Zheng et al. in 2020.^109^ In the tumor microenvironment, reductive GSH, one of the biothiols that is overexpressed in cancer, can release H_2_S from DATS, and consequently the released H_2_S was used for either gas therapy or reduction of azide (−N_3_ (−)) to ammonium ion (−NH_3_ (+)), promoting PTT together with PA due to prolonged tumor retention. Under 808 nm laser irradiation, the temperature was increased in a dose dependent manner, and AB-DS@BSA-N_**3**_ exhibited selective photothermal toxicity in Hep2 cells with slight toxicity in control groups. *In vivo* studies showed that while the tumor can be monitored successfully through PA or fluorescence channels using AB-DS@BSA-N_**3**_, the tumor was completely suppressed after NIR laser irradiation in Hep2 tumor-bearing mice.

**Figure 21:**
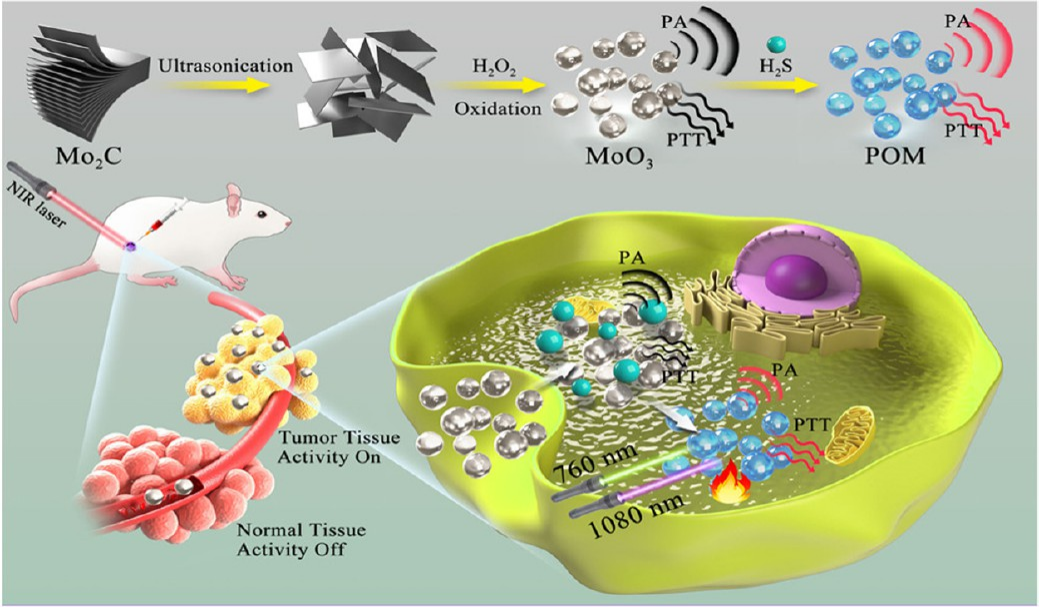
Schematic depiction of the preparation of the MoO_3_ nanoparticles and their activation of PA and PTT channels in the presence of endogenous H_2_S. Adapted with permission from ref (110). Copyright 2021 American Chemical Society.

In 2021, H_2_S responsive water-soluble MoO_3_ nanoparticles were designed and developed by Wang et al. for PTT and PA imaging.^110^ The nanoparticles were prepared with an average size of 9 nm through a one-pot process encompassing ultrasonication and oxidation (Figure 21). Initially, MoO_3_ nanoparticles remained in their off-state, but addition of NaHS generated polyoxometalates (POMs) via redox reaction (Figure 21) and activated both NIR-I and NIR-II channels at 760 and 1080 nm in a dose dependent manner. A similar trend was recorded in thermal and PA channels. In good correlation with the characterization, MoO_3_ nanoparticles displayed photothermal cytotoxicity in HCT116 and 4T1 cells after laser irradiation. Also, PTT was activated by endogenous H_2_S in HCT116 tumor-bearing mice and resulted in suppression of the tumor. Photothermal conversion efficiency was determined to be higher with NIR-II irradiation as survival rate was lower in NIR-II irradiation in both cases. Additionally, PA images were captured successfully through both channels. Similarly, the signal-to-noise ratio was higher in the NIR-II region.

**Figure 22:**
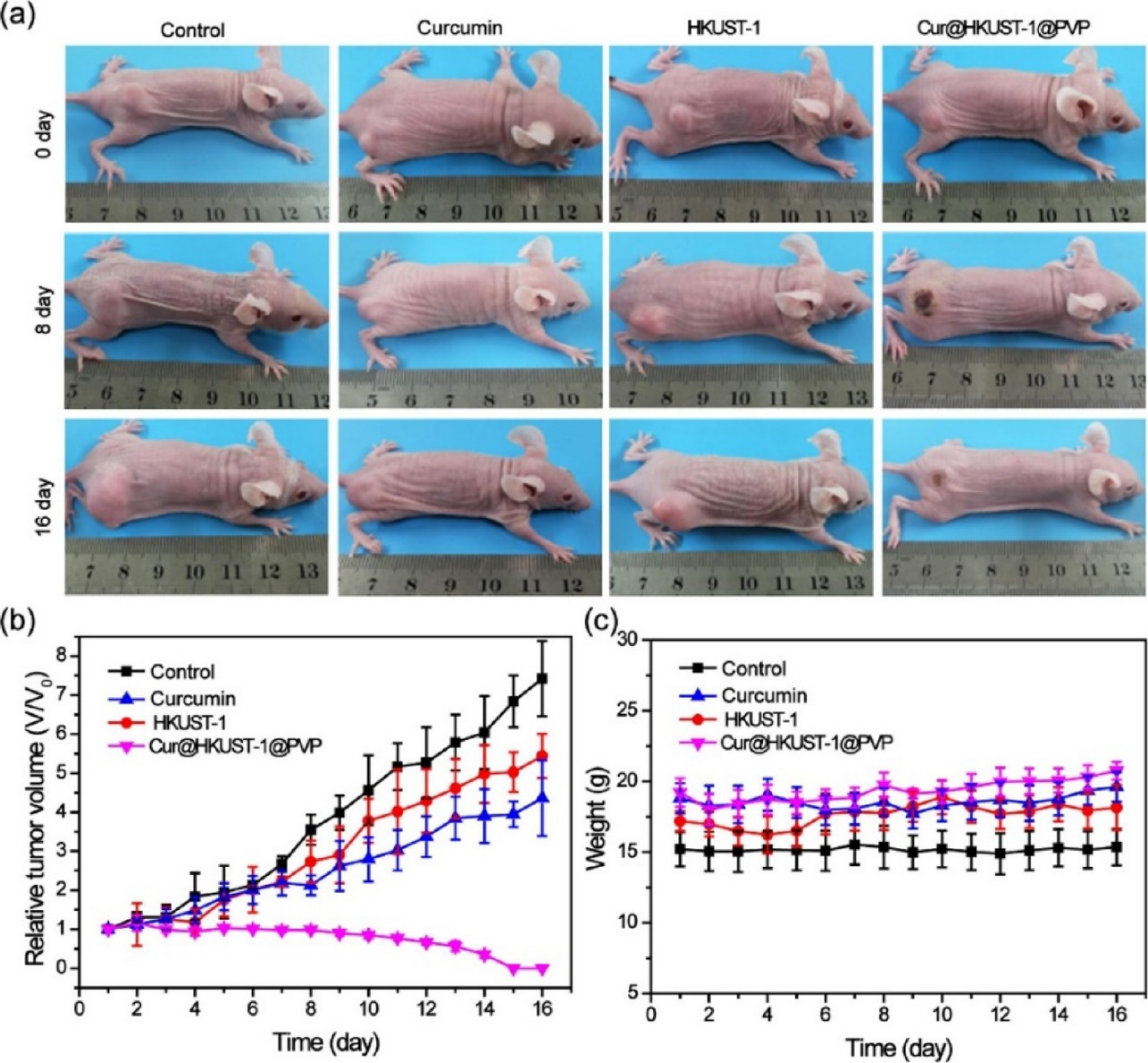
(a) Photographs of HCT116 tumor bearing mice captured before and at 8 and 16 days after the treatment. First row: control; Second row: Curcumin; Third row: HKUST-1; Fourth row: Cur@HKUST-1@PVP. (b) Evaluation of the tumor progression captured over the course of 16 days. (c) Body weights of the mice groups during the therapeutic action. Adapted with permission from ref (111). Copyright 2022 Elsevier.

A MOF that consisted of trimesic acid, copper, and curcumin (Cur@HKUST-1@PVP) was first introduced by Tian et al. in 2022 to treat colon cancer cells by combining PTT and chemotherapy.^111^ During the synthesis of this therapeutic agent, curcumin was loaded into the HKUST-1 network, and the improper release of curcumin was blocked with the surface ligand polyvinylpyrrolidone (PVP). Upon reaction with gradually increasing concentrations of H_2_S, the absorption intensity of Cur@HKUST-1@PVP at 980 nm was shown to increase. It was also demonstrated that the photoacoustic intensity of H_2_S-treated Cur@HKUST-1@PVP displayed a 5-fold increase compared to the control groups, and upon irradiation of the NaHS incubated cores, a temperature rise (10–21 °C) was observed. The *in vivo* studies of Cur@HKUST-1@PVP demonstrated the colon cancer cell selectivity of the probe as the same 5-fold increase in photoacoustic intensity was only observed at tumor sites. Following its irradiation, the probe was also reported to completely eradicate the colon tumor cells in 16 days *in vivo*, while HKUST-1 and curcumin control groups only managed to suppress tumor growth (Figure 22).

## 4 Conclusion and Outlook

To close, we summarize here the recent advances in H_2_S-responsive PDT and PTT agents by covering both small organic molecule and nanoparticle based designs (Table 1).

**Table 1:** Summary of H_2_S Responsive Phototherapy Agents

name	abs/ems (nm)	cell line	in vivo	therapy	ref
MOF NP-1	420/610–660	HepG2, HCT116 LoVo	mice	PDT	(95)
1^2+^-SNP830-FA	500–850/830	RAW264.7, KB, HCT116, HT29, HEK293	mice	PDT	(96)
Nano-TNP-SO	655/712	HepG2, HCT116	mice	PDT	(97)
TDBP	422/660	HCT116, LO2, CT26, HepG2	none	PDT	(98)
DB2T	534/579	HepG2, HCT116, PC12, HUH-7D	mice, zebrafish	PDT	(99)
NP-Cu	400/660 and 750–1000/nd	HCT116, HepG2 HeLa	mice	PDT, PTT, CDT	(100)
TDCAc	575/nd	AGS	mice	PDT	(101)
ZNNPs	680/720	HCT116, HEK293	mice	PDT	(102)
RHS	586/606	SH-SY5Y, L929	none	PDT	(103)
3RAX-NBD	540/610	4T1, HeLa MCF-7	mice	PDT	(93)
Cu_2_O	700–1000/nd	HCT116, HUVEC	mice	PTT	(104)
nano-PT	790/900–1300	HCT116, HepG2	mice	PTT	(105)
lnTBOD-Cl	756/900	HCT116, HepG2	mice	PTT	(106)
Au@Cu_2_O	800–1000/nd	HCT116, HUVEC	mice	PTT	(107)
EA-Fe@BSA	450–900/nd	HCT116, HUVEC RBC	mice	PTT, CDT	(108)
AB-DS@BSA-N_3_	300–900/1000–1400	Hep2	mice	PTT, gas therapy	(109)
MoO_3_-NP	760–1080/nd	HCT116, 4T1 HL-7702	mice	PTT	(110)
Cur@HKUST-1@PVP	700–1000/nd	HCT116, HUVEC	mice	PTT	(111)

It is a well-established fact that an abnormal level of H_2_S is directly associated with a wide variety of pathological states. Thus, it appears as an attractive biomarker that can be utilized in activity-based phototherapy applications. As evidenced from the current literature examples presented in this review, there is a growing interest toward development of H_2_S-responsive and multifunctional phototherapy agents. However, some critical limitations such as off-target activation of H_2_S activatable PSs, phototoxicity of the caged PSs, high reactivity of H_2_S with other biological molecules due to its high nucleophilicity and redox activity, and interference of other endogenous biothiols still need to be addressed. There are also some other drawbacks arising from the chronic problems of phototherapies such as low cytotoxicity of single mode PTT agents, poor performance of PSs in aqueous environments and under hypoxic conditions, and limited penetration of the excitation light through tissues. Although recent advances in the field of both small molecule and nanomaterial based PSs offer judicious solutions to these challenges, there is still room for development to improve the therapeutic outcome. In this direction, we anticipate that new H_2_S-responsive agents that can combine phototherapy and other modes of therapeutic actions such as chemotherapy, radiotherapy, and sonodynamic therapy will start to increase in the coming years, which will improve the efficacy of the therapy. Additionally, one should expect that these multimodal agents may also enable precise imaging by utilizing fluorescence in the NIR II region and PA, magnetic resonance (MR), or positron emission tomography (PET) techniques. Furthermore, there is no doubt that organelle targeted photosensitizers are promising candidates to achieve high cytotoxicity in phototherapy applications. It is very likely for a H_2_S-responsive agent to be activated in normal cells, as H_2_S also plays important roles in physiological processes. To this end, new generation H_2_S-responsive agents should have improved selectivity. This can be achieved by dual-locked agents, which can be activated in the presence of H_2_S and another related biomarker. Furthermore, it should not be surprising to see more examples soon where H_2_S itself is utilized as a cytotoxic agent. Finally, special attention needs to be paid to the pharmacokinetics and pharmacodynamics characteristics of H_2_S activatable agents in animal models to pave the way for clinical translation.
